# Supplementary material for: Perioperative morbidity and mortality of cardiothoracic surgery in patients with a do-not-resuscitate order
Source: PeerJ. 2014 Jan 22;2:e245. doi: 10.7717/peerj.245 (PMC3912447; doi:10.7717/peerj.245)
Supplement: Appendix [file peerj-02-245-s001.docx]

Appendix: Procedure codes used to define cardiac surgery

| ICD-9-CM Code | Procedure |  |
| --- | --- | --- |
| 35.1 | Open heart valvuloplasty without replacement |  |
| 35.11 | Open heart valvuloplasty of aortic valve without replacement |  |
| 35.12 | Open heart valvuloplasty of mitral valve without replacement |  |
| 35.14 | Open heart valvuloplasty of tricuspid valve without replacement |  |
| 35.21 | Open and other replacement of aortic valve with tissue graft |  |
| 35.22 | Open and other replacement of aortic valve |  |
| 35.23 | Open and other replacement of mitral valve with tissue graft |  |
| 35.24 | Open and other replacement of mitral valve |  |
| 35.26 | Open and other replacement of pulmonary valve |  |
| 35.27 | Open and other replacement of tricuspid valve with tissue graft |  |
| 35.28 | Open and other replacement of tricuspid valve |  |
| 35.3 | Operations on structures adjacent to heart valves |  |
| 35.32 | Operations on chordae tendineae |  |
| 35.33 | Annuloplasty |  |
| 35.42 | Creation of septal defect in heart |  |
| 35.51 | Repair of atrial septal defect with prosthesis, open technique |  |
| 35.52 | Repair of atrial septal defect with prosthesis, closed technique |  |
| 35.53 | Repair of ventricular septal defect with prosthesis, open technique |  |
| 35.55 | Repair of ventricular septal defect with prosthesis, closed technique |  |
| 35.61 | Repair of atrial septal defect with tissue graft |  |
| 35.62 | Repair of ventricular septal defect with tissue graft |  |
| 35.63 | Repair of endocardial cushion defect with tissue graft |  |
| 35.71 | Other and unspecified repair of atrial septal defect |  |
| 35.72 | Other and unspecified repair of ventricular septal defect |  |
| 35.81 | Total repair of tetralogy of fallot |  |
| 35.82 | Total repair of total anomalous pulmonary venous connection |  |
| 35.83 | Total repair of truncus arteriosus |  |
| 35.9 | Other operations on valves and septa of heart |  |
| 35.92 | Creation of conduit between right ventricle and pulmonary artery |  |
| 35.93 | Creation of conduit between left ventricle and aorta |  |
| 35.94 | Creation of conduit between atrium and pulmonary artery |  |
| 35.96 | Percutaneous balloon valvuloplasty |  |
| 35.99 | Other operations on valves of heart |  |
| 36.11 | (Aorto)coronary bypass of one coronary artery |  |
| 36.12 | (Aorto)coronary bypass of two coronary arteries |  |
| 36.13 | (Aorto)coronary bypass of three coronary arteries |  |
| 36.14 | (Aorto)coronary bypass of four or more coronary arteries |  |
| 36.15 | Single internal mammary-coronary artery bypass |  |
| ICD-9-CM Code | Procedure | |
| 36.16 | Double internal mammary-coronary artery bypass | |
| 36.31 | Open chest transmyocardial revascularization | |
| 36.32 | Other transmyocardial revascularization | |
| 36.91 | Repair of aneurysm of coronary vessel | |
| 36.99 | Other operations on vessels of heart | |
| 37.1 | Incision of heart, not otherwise specified | |
| 37.11 | Cardiotomy | |
| 37.12 | Pericardiotomy | |
| 37.24 | Biopsy of pericardium | |
| 37.25 | Biopsy of heart | |
| 37.31 | Pericardiectomy | |
| 37.32 | Excision of aneurysm of heart | |
| 37.33 | Excision or destruction of other lesion or tissue of heart, open approach | |
| 37.34 | Excision or destruction of other lesion or tissue of heart, other approach | |
| 37.49 | Other repair of heart and pericardium | |
| 37.51 | Heart transplantation | |
| 37.61 | Implant of pulsation balloon | |
| 37.62 | Insertion of temporary non-implantable extracorporeal circulatory assist device | |
| 37.63 | Repair of heart assist system | |
| 37.64 | Removal of external heart assist system(s) or device(s) | |
| 37.65 | Implant of single ventricular (extracorporeal) external heart assist system | |
| 37.66 | Insertion of implantable heart assist system | |
| 37.68 | Insertion of percutaneous external heart assist device | |
| 38.04 | Incision of vessel, aorta | |
| 38.05 | Incision of vessel, other thoracic vessels | |
| 38.14 | Endarterectomy, aorta | |
| 38.15 | Endarterectomy, other thoracic vessels | |
| 38.34 | Resection of vessel with anastomosis, aorta | |
| 38.35 | Resection of vessel with anastomosis, other thoracic vessels | |
| 38.44 | Resection of vessel with replacement, aorta, abdominal | |
| 38.45 | Resection of vessel with replacement, thoracic vessels | |
| 38.64 | Other excision of vessels, aorta, abdominal | |
| 38.84 | Other surgical occlusion of vessels, aorta, abdominal | |
| 38.85 | Other surgical occlusion of vessels, thoracic vessels | |
| 39.0 | Systemic to pulmonary artery shunt | |
| 39.21 | Caval-pulmonary artery anastomosis | |
| 39.22 | Aorta-subclavian-carotid bypass | |
| 39.54 | Re-entry operation (aorta) | |
| 39.64 | Intraoperative cardiac pacemaker | |
| 39.65 | Extracorporeal membrane oxygenation | |
